# Protective effects of selenium on electromagnetic field-induced apoptosis, aromatase P450 activity, and leptin receptor expression in rat testis

**DOI:** 10.22038/ijbms.2021.45358.10554

**Published:** 2021-03

**Authors:** Sareh Khoshbakht, Fatemeh Motejaded, Sareh Karimi, Narjes Jalilvand, Alireza Ebrahimzadeh-Bideskan

**Affiliations:** 1 Department of Anatomy and Cell Biology, School of Medicine, Mashhad University of Medical Sciences, Mashhad, Iran; 2 Department of Biology and Anatomical Sciences, School of Medicine, Shahid Beheshti University of Medical Sciences, Tehran, Iran; 3 Applied Biomedical Research Center, Mashhad University of Medical Sciences, Mashhad, Iran

**Keywords:** Apoptosis, Electromagnetic radiation, Leptin receptor, Selenium, Testis

## Abstract

**Objective(s):**

Electromagnetic field (EMF) emitted by mobiles may affect the male reproductive system. Selenium, as an antioxidant, may protect against electromagnetic field-induced tissue damage. Theis study aimed to investigate the effects of selenium on rat testis exposed to electromagnetic fields.

**Materials and Methods:**

Twenty-four male Wistar rats were divided into four groups, namely EM group (2100 MHZ), EM/SE group (2100 MHZ + selenium (0.2 mg/kg), SE group (selenium 0.2 mg/kg), CONT (control group). Serum LH, FSH, testosterone, leptin and aromatase levels, testis weight and volume index, sperm parameters (count and abnormal percent), seminiferous tubule diam¬eters, germinal epithelia thickness, immunoreactivity of leptin receptor and caspase-3 (for apoptotic cells in germinal epithelium) were investigated.

**Results:**

Our results showed that serum LH, FSH, GnRH, testosterone level, sperm count, germinal epithelium thickness, and seminiferous tubule diameter were significantly declined in the EM group compared with the CONT group (*P*<0.05). However, in the EM group, the serum leptin level, sperm abnormality, aromatase enzyme level, apoptotic cells, and leptin receptor were increased compared with the CONT group (*P*<0.05). Furthermore, an increase in sperm count, germinal epithelium thickness, seminiferous diameters, serum LH, FSH, and GnRH, and testosterone levels, and a significant decrease in sperm abnormality, leptin receptor and apoptotic cells in the EM/SE group compared with the EM group were also observed (*P*<0.05).

**Conclusion:**

This study showed that electromagnetic radiation may have detrimental impacts on the male reproductive system, which can be prevented by use of selenium.

## Introduction

Over the recent decades, the use of handheld wireless communication devices such as cell phones has been rapidly increasing, hence the increase in public concern as to the health risks of the radiofrequency created by these technologies (1). During use, smartphones emit radiofrequency radiation, a non-ionizing radiation with a range of 800 MHZ to 4 GH (2). According to previous studies, cell phone waves can have adverse effects on the cardiovascular system (3), immune system (4), thyroid and its hormone secretion (5), female reproductive system (6), and malignant tumors (7). The effects of these radiations are partly observed in the male reproductive system, including damage to the testicular tissue through the formation of apoptosis in seminiferous tubules (8), decrease in the diameter of seminiferous tubules (9), delay in puberty, changes in semen parameters (10), and reduction in serum testosterone levels (11).

Discovered by Jöns Jacob Berzelius in 1817, selenium is an essential element in maintaining the health of mammals. Selenium compounds such as selenocysteine in the selenoprotein molecule, play a structural and enzymatic role in the body (12). Some studies have shown that this element is able to protect the cell membranes from the harmful effects of oxidative stress (12). Selenium is essential for the proper function of the immune system (13) and plays catalytic roles in the secretion of thyroid hormones (14). Among the key functions of selenium, mention can be made of reducing the risk of abortion (via reducing oxidative stress, endoplasmic stress, and vascular tone regulation) (15), preventing the development of viral diseases (16), decreasing pancreatitis (17) and treating ovarian cysts in women through positive effects on insulin metabolism and VLDL level (18). Concerning the male reproductive system, selenium prevents mitophagy in Sertoli cells, controls the immune genes and proteins existing in the blood-testis-barrier (19), increases the secretion of testosterone from Leydig cells and the diameter of the seminiferous tubules (20), and improves morphology and sperm motility (20). Selenium may reduce apoptosis in testicular tissues by controlling the oxidative stress and increasing anti-apoptotic proteins (such as bcl2) (20).

Leptin is mainly secreted by white adipose tissue (21) and it is critical for normal sexual function. Some of the previous studies have suggested that excessive leptin secretion may detrimentally affect sperm parameters. Overweight males have high leptin serum levels and there exists a positive correlation between high leptin and sperm dysfunction (22, 23). Leptin receptor belongs to class I cytokine superfamily, in which the hypothalamus, pancreas, kidneys, spermatozoa, testis, and skeletal muscles are present (24). 

It is necessary to study the pathways and enzymes that cause estrogen production in men. One of the vital enzymes involved in this pathway is cytochrome p450 aromatase (CYP19), which irreversibly converts androgenic hormones to estrogens. Aromatase is found in several parts of the body, including the brain, adipose tissue, bones, heart, Sertoli cells, germ cells, and Leydig cells (25, 26). 

This research aimed to assess the selenium effects on electromagnetic field-induced apoptosis, leptin receptor expression, and aromatase P450 enzyme activity in testicular tissue, sexual hormone production, and sperm parameter changes in rats.

## Materials and Methods

The Ethics Committee for the Care and Use of Lab Animals at Mashhad University of Medical Sciences (IR.MUMS.MEDICAL.REC.1397.461) controlled the present study.

***Electromagnetic field (EMF)***

**Figure 1:**
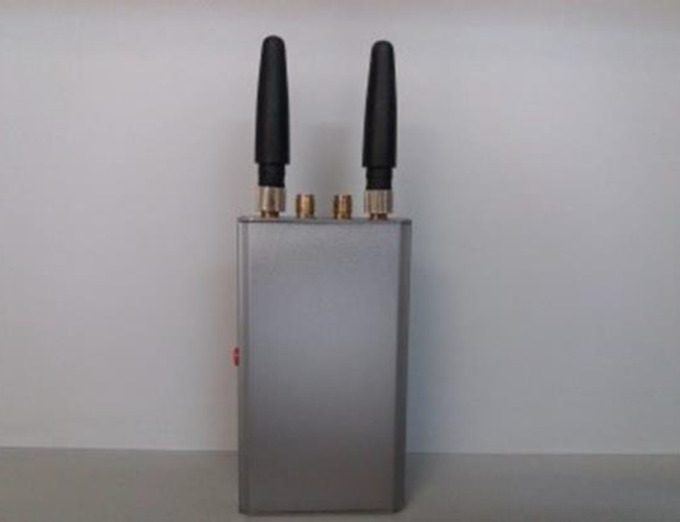
The device that was used for Electromagnetic field generation

The electromagnetic field was generated with a tool producing 2100 MHZ, which is equivalent to 4G-LTE cell phones (Figure 1). 

***Animals***

In the current study, 24 Wistar rats (64 days old) (were used, which were obtained from the Animal Lab of Mashhad University of Medical Sciences, Mashhad, Iran. The rats were kept under standard conditions (24 hr cycle: 12 hr day and 12 hr night, temperature: 22±2 °C, and 50% relative humidity).

The rats were randomly assigned into four groups:

1) EM group: the rats were exposed to EMF (2100 MHZ) for 2 hr daily, then injected with distilled water (1 ml/100 g of body weight) for 16 days (27). 

2) EM+SE group: the rats were exposed to EMF (2100 MHZ) for 2 hr daily, then injected with selenium (0.2 mg/kg per day) for 16 days (28).

3) SE group: the rats were administered selenium (0.2 mg/kg per day) for 16 days (28). 

4) CONT group: the rats were kept without any intervention.

***Biochemical analyses***

At the end of the experiments (17^th^ day), all rats were anesthetized by ether and the blood sampling was done to separate the serum with centrifugation and stored at -20 °C. The concentrations of gonadotropin-releasing hormone (GnRH), hormone-stimulating follicle (FSH), luteinizing hormone (LH), testosterone, leptin, and aromatase enzyme were evaluated using the enzyme-linked immunosorbent assay (ELISA) method under the manufacturer’s provided instructions (29). 

***Volume and weight index of the testis***

The left testes were taken for volume and weight measurements and histological examinations. Testes weight index and volume were calculated according to the following formulas

$\mathrm{Wi}=\frac{\mathrm{Testis weight}(\mathrm{gr})}{\mathrm{Animal body weight}(\mathrm{gr})}\times 100$

Wi= weight index 

$\mathrm{Vt}=\frac{\left(\mathrm{W2}-\mathrm{W1}\right)}{\delta }$

Vt= Weight of testis 

W1 = Primary weight (basket+ normal saline + Beaker)

W2 = Secondary weight (basket + normal saline + Beaker + testis)

δ = Normal saline density (1.0048) (30) 

***Immunohistochemistry methods***

The removed testes were fixed in 10% neutral buffer formalin (NBF) for four days. Following fixation, the specimens were prepared according to histology laboratory routine methods, embedded in paraffin, and then cut into 5 µm thickness using a Leitz microtome. 

*Leptin receptor*

Tissue sections were deparaffinized in xylene, rehydrated by descending grades of alcohol, and then washed with PBS. Antigen retrieval was done using protein kinase, and endogenous peroxidase was neutralized with 3% H_2_O_2_ in ethanol for 15 min and then rinsed in goat serum. Finally, the samples were incubated with leptin receptor antibody (catalog number = orb318718/ concentration= 0.5 mg/ml) at 4 °C overnight. They were washed with PBS and incubated with a secondary antibody in a humid chamber at room temperature for 3 hr. Afterward, the sections were extensively washed with PBS and covered with DAB solution (0.03 g DAB dissolved in 100 ml of PBS and 200 µl H_2_O_2_/100 ml PBS) for 15 min at room temperature in the dark. The samples were washed in running water then were counterstained with 1% hematoxylin solution for 20 min. Finally, for dehydration, tissue sections were placed in increasing ethanol grades, passed through xylene, and mounted in a glass slide. In the glass slides, the cells with brown cytoplasm were appraised as leptin receptor-positive.

*Caspase-3*

All the above-mentioned steps for leptin Receptor immunohistochemistry were further applied to caspase-3 antibodies with different types of tested antibodies. For the localization of caspase-3 , sections were incubated with anti-caspase-3 antibody (catalog number =Orb10237/concentration= 0.2 mg/ml) at 4 °C overnight. Next, tissue sections were washed and finally incubated with secondary antibody (catalog number = Orb43514/ concentration= 1 mg/ml) in the humid chamber for 3 hr at room temperature (31).

*Staining intensity evaluation*

To evaluate the immunoreactivity of tissue sections, ten images were taken from each section using a light microscope (Olympus BX51, Japan). Sections were graded by 3 blind examiners according to Likert spectrum (Based on the staining intensity; feeble reaction = +‏, modest reaction = ++, severe reaction = +++ and very severe reaction = ++++) (32). 

***Seminiferous tubules diameter and germinal epithelium height***

To calculate the diameter of the seminiferous tubules, sections were taken transversely from the testes, ten images were randomly taken from each slide using ×40 objective lens of a microscope equipped with a great-resolution camera (dp12). The taken photos were transferred to the computer and an image processing software was employed to measure the seminiferous diameter and germinal epithelium thickness. 

***Sperm count***

The left epididymis was removed, cut into 5 parts, immersed in 5 ml of normal saline, and then incubated at 37 °C for 15 min. The sperms in each sample were counted by using Neobar slide and 40x objective lens of a light microscope. The sperms in four squares were numbered and the total sperm count was calculated according to the following formula (33):

$N=\frac{\mathrm{mean of sperm counted in 4 square}\times \mathrm{delution}(5)}{\mathrm{A square volume}(\mathrm{nl})}$

***Sperm morphology***

To assess sperm morphology, the epididymis sperm solution was smeared on a slide (50 µl), fixed with methanol (70%), and finally stained with toluidine blue. Using an optical microscope, 200 sperms were counted in each slide, and the sperms without tails, with coiled or bent tails, or dual or abnormal heads were recognized as having abnormal morphology (34).

***Statistical analysis***

To compare all the studied groups, SPSS statistic software (ver. 16), one-way ANOVA, Tukey’s *post hoc*, Kruskal–Wallis, and Mann–Whitney tests were used for parametric and non-parametric statistical tests, respectively. Values were expressed as means ± standard error of the mean (SEM) and *P-values* less than 0.05 were assumed as statistically significant.

## Results

***Biochemical analyses***

The GnRH serum level significantly decreased in the EM group (*P*≤0.05) and increased in the SE group (*P*≤0.05) compared with the CONT group. Also, in the EM/SE group, the GnRH serum level significantly increased in comparison with the EM group (*P*≤0.05). 

 Although the serum level of LH and FSH were significantly reduced in the EM group (*P*≤0.05), in the SE group it increased in comparison with the CONT group (*P*≤0.05). In the EM/SE group, both hormones (LH and FSH) were higher than in the EM group, significantly (*P*≤0.05).

Our results showed a significant decrease in testosterone serum levels in the EM group and an increase in the SE group compared with the CONT group (*P*≤0.05). A significant increase in levels of testosterone was detected in the EM/SE group compared with the EM group (*P*≤0.05).

Serum aromatase level in the EM/SE group was lower than in the EM group, significantly (*P*≤0.05). In addition, serum leptin levels significantly increased in the EM group, and decreased in the SE group in comparison with the CONT group (*P*≤0.05); moreover, leptin hormone in the EM/SE group was lower than in the EM group (*P*≤0.05).

***Testis weight and Testes volume***

The mean of testicular weight index showed a decrease in the EM group, increase in the SE and EM/SE groups compared with the control group. However, these differences were not significant. Testicular volume was reduced significantly in EM and increased in SE group. Comparison of EM/SE and EM groups showed that the testicular volume increased in EM/SE though not significantly.

***Immunohistochemistry***

*Leptin receptor*

The findings of this study indicated that leptin expression was reduced in all studied spermatogenic cells (spermatid, primary spermatocyte, and spermatogonia) in the SE group compared with the CONT group (*P*≤0.05). The leptin receptor expression was higher in the EM group than in the CONT group (spermatogonia, primary spermatocyte, and spermatid: *P*≤0.05). Moreover, the expression of these receptor spermatogenic cells was lower in the EM/SE group compared with the EM group (*P*≤0.05).

*Caspase-3*

The expression of caspase-3 was enhanced in the EM group compared with the CONT group in all germinal epithelium cells including spermatogonia (*P*≤0.05), primary spermatocyte (*P*≤0.05), and spermatid (*P*≤0.05) and reduced in the SE group in comparison with the CONT group (spermatogonia (*P*≤0.05), primary spermatocyte (*P*≤0.05), and spermatid (*P*≤0.05). Caspase-3 expression in all the studied cells was reduced in the EM/SE group in comparison with the EM group (spermatogonia, primary spermatocyte, and spermatid, *P*≤0.05).

***Histological measurements***

The diameter of the seminiferous tubules was decreased in the EM group (*P*≤0.05) and increased in the SE group in comparison with the CONT group (*P*≤0.05). Tubular diameter in EM/SE group was more than in the EM group (*P*≤0.05). 

Similar results were obtained regarding the germinal epithelium thickness measurements, where a significant reduction in the epithelium was found in the EM group versus the CONT group (*P*≤0.05); germinal epithelium in the SE group was more than in the CONT group (*P*≤0.05). An increase in diameter occurred in the EM/SE group compared with the EM group, significantly (*P*≤0.05).

***Sperm parameters***

Our results indicated that the sperm count was reduced in the EM group (*P*≤0.05) and enhanced in the SE group compared with the CONT group (*P*≤0.05). Also, there was an increase in the number of sperm in the EM/SE group compared with the EM group (*P*≤0.05).

The percentage of abnormal sperm was higher in the EM and lower in the SE group compared with the CONT group (*P*≤0.05). The percentage of abnormal sperm in the EM/SE group was lower than in the EM group (*P*≤0.05).

**Figure 2:**
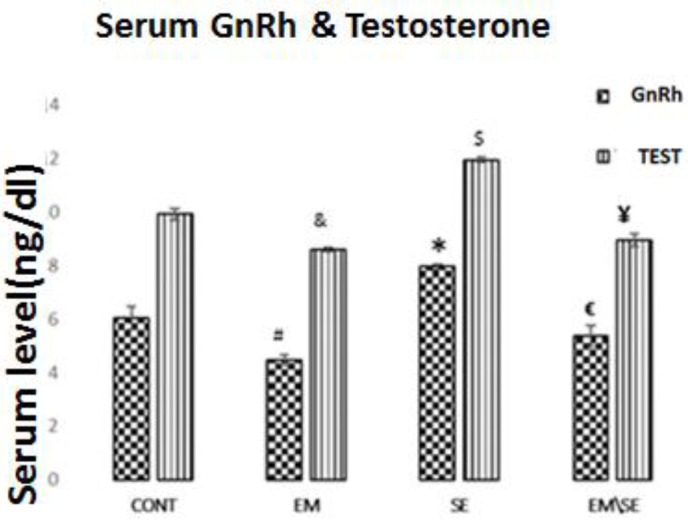
Comparison of GnRH serum levels in different studied groups. GnRH reduced in EM group in comparison with the CONT group (^#^*P*≤0.05), enhanced in the SE group compared with CONT group (*P≤0.05), and increased in EM/SE group in comparison with EM group (^€ ^*P*≤0.05). Serum testosterone level in the EM group was significantly lower than in the CONT group (^&^*P*≤0.05), and in the SE group was much more than the CONT group (^$^*P*≤0.05). Also, serum testosterone levels enhanced in EM/SE group compared with the EM group (^¥^*P*≤0.05)

**Figure 3:**
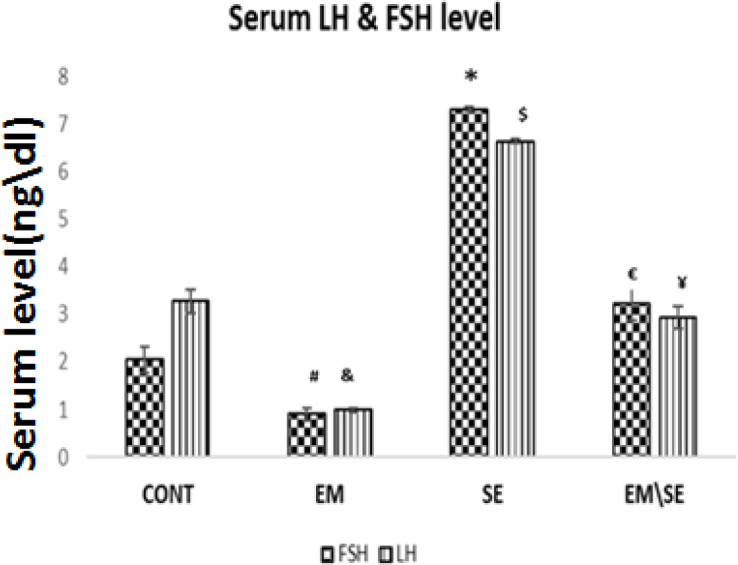
Comparison of serum LH and FSH levels in studied groups. Both hormones significantly decreased in the EM group (^# &^*P*≤0.05) and enhanced in the SE group compared with the CONT group (^$^ *P≤0.05). Serum LH and FSH levels in the EM/SE group were more than in the EM group, significantly (£ ¥*P*≤0.05)

LH: luteinizing hormone; FSH: hormone-stimulating follicle

**Figure 4:**
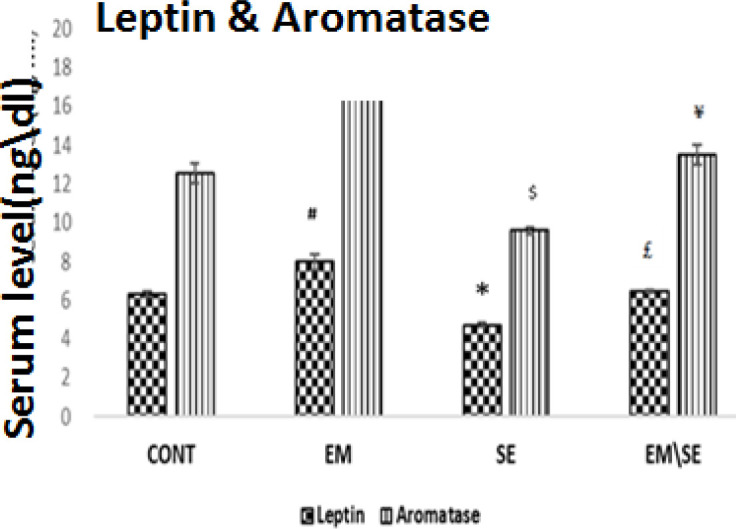
Evaluation of the mean of serum aromatase enzyme indicated that this enzyme in EM group was more than in the CONT group (^&^ *P*≤0.05), in the SE group was lower than in the CONT group (^$ ^*P*≤0.05), and significantly reduced in the EM/SE group compared with EM group (^¥^ *P*≤0.05)

The mean serum leptin level was significantly enhanced in the EM group (^#^*P*≤0.05) and reduced in the SE group (* *P*≤0.05) in comparison with the CONT group. Serum leptin level significantly decreased in EM/SE group compared with the EM group (^£^*P*≤0.05)

**Figure 5:**
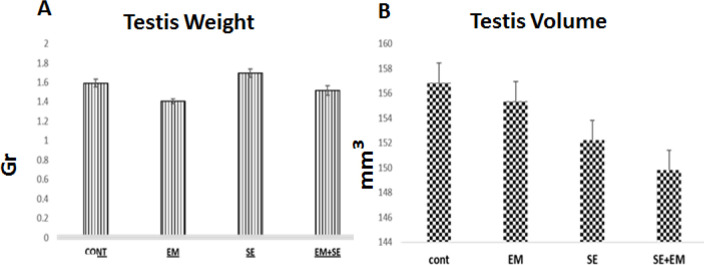
A & B. Comparison of the mean weight index and mean of testis volume did not show a significant difference between groups

**Figure 6:**
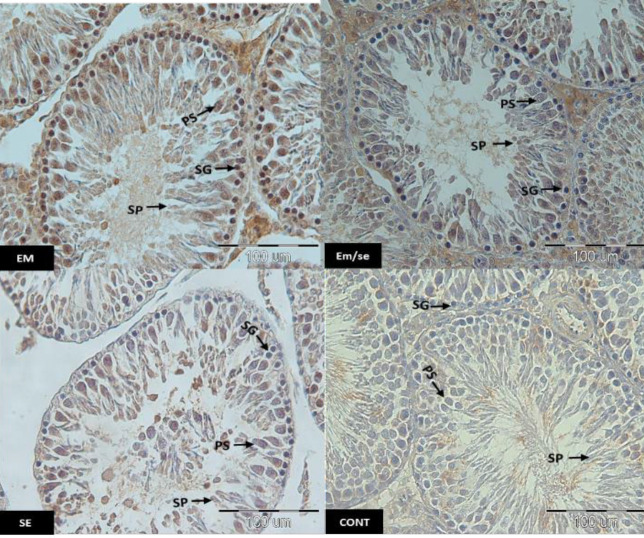
Immunostaining of the testicular tissue sections incubated with anti leptin receptor. The arrows represent SG: spermatogonia, PS: primary spermatocytes, SP: spermatids, EM=electeromagnetic field, SE= selenium, CONT= Control, scale bar= 100 µm

**Figure 7:**
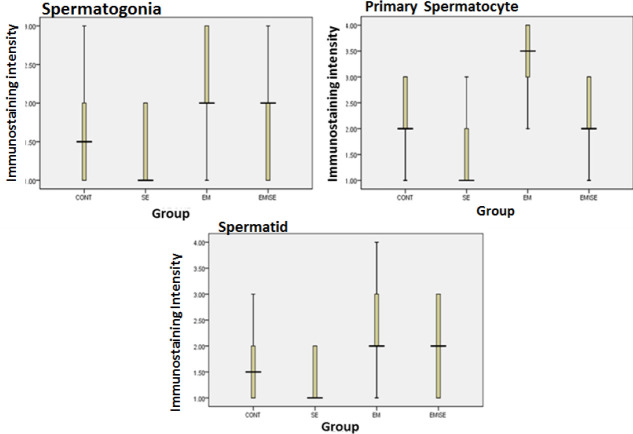
Comparison of leptin receptor immunoreactivity, leptin receptor immunoreactivity in the EM group was more than in the CONT group (spermatogonia, primary spermatocyte, and spermatid, *P*≤0.05). There was a decrease of immunoreactivity in the SE group compared with the CONT group (spermatogonia, primary spermatocyte, and spermatid, *P*≤0.05) and immunoreactivity reduction in EM/SE compared with EM (primary spermatocyte and spermatid: *P*≤0.005)

**Figure 8:**
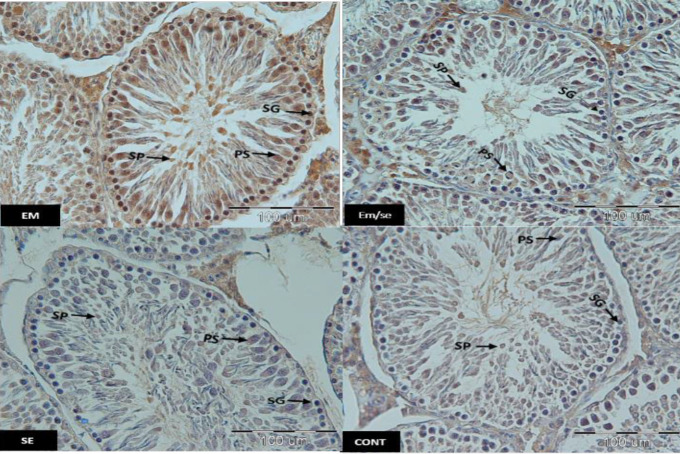
Immunostaining of testicular tissue sections incubated with anti caspase-3 antibody in studied groups. SP: spermatids, PS: primary spermatocytes, SG: spermatogonia, EM= electromagnetic field, SE= selenium, CONT= control, scale bar= 100 µm

**Figure 9:**
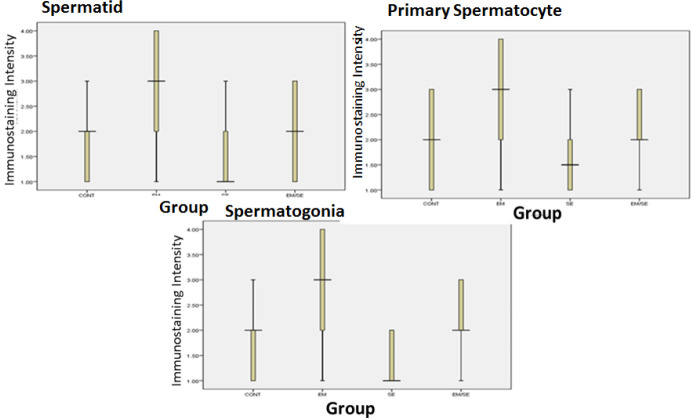
Comparison of caspase-3 immunoreactivity between different studied groups indicated an increase in the EM group in comparison with the CONT group (spermatogonia, primary spermatocyte, and spermatid, *P*≤0.05), a decrease in SE group compared with the CONT group (spermatogonia, primary spermatocyte, and spermatid, *P*≤0.05) and a reduction in EM/SE in comparison with the EM group (spermatogonia, primary spermatocyte, and spermatid, *P*≤0.05). These changes were observed in all germinal cell lines (spermatogonia, primary spermatocyte, and spermatid)

**Figure 10:**
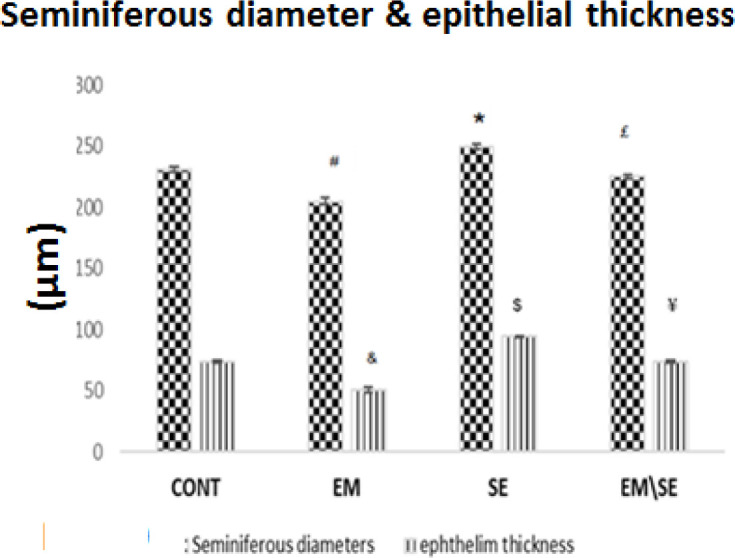
By comparison of the mean of seminiferous tubule diameters, a decrease was indicated in the EM group (^#^*P*<0.05) and an increase in the SE group (^*^P<0.05) compared with the CONT group. These changes also significantly increased in the EM/SE group compared with the EM group (^£^*P*<0.05). In addition, germinal epithelium thickness was reduced in the EM group (^&^*P*<0.05) and increased in the SE group (^$^*P*<0.05) compared with the CONT group and significantly increased in EM/SE group compared with the EM group (^¥^ *P*<0.05)

**Figure 11:**
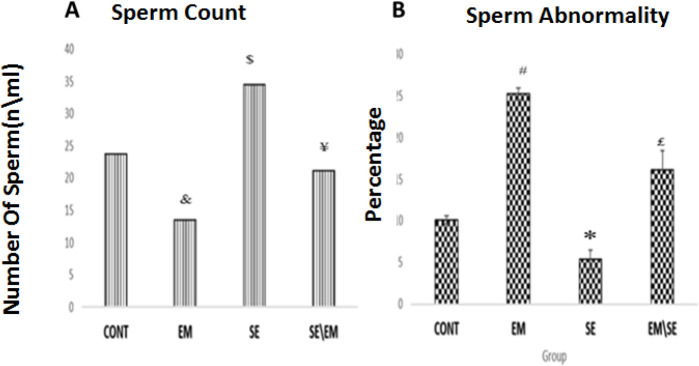
B. Statistical analysis of counted sperm showed that the number of sperm was decreased in the EM group (&*P*≤0.05) and increased in the SE group versus the CONT group, significantly (^$^ *P*≤0.05). These alterations in the EM/SE group were also higher than in the EM group (¥ *P*<0.05), (Figure 11A). Abnormal sperm percentage increased in the EM group (^#^ *P*≤0.05), decreased in the SE group in comparison with the CONT group (^*^ *P*≤0.05). Finally, Abnormal sperm percentage was decreased in the EM/SE group versus the EM group (^£^ *P*≤0.05)

## Discussion

Although cell phones facilitate communications and information access, the produced electromagnetic waves impact the living cells and tissues (3, 5). It has been reported that these waves are able to reduce the diameter of the seminiferous tubules, the germinal epithelium thickness, and sperm count (9, 10).

 As a natural antioxidant, selenium is involved in the formation of various enzymes including glutathione peroxidase and thioredoxin reductase which eliminate free radicals, thereby protect the tissue against oxidative degradation (12, 20).

In the current investigation, in the EM group, the concentrations of testosterone, LH, FSH, and GnRH decreased significantly. Previous studies have shown cell phone radiation reduces hormones released in the hypothalamic-pituitary-gonad axis (35). Moreover, rats undergoing 2-hr electromagnetic exposure over a 60 day period had lower LH, FSH, and GnRH serum levels than the CONT group (36). However, some studies have claimed that the electromagnetic field is not able to change serum FSH levels (37). Such discrepancy might stem from the different electromagnetic field intensities utilized in different experiments.

The decline in GnRH, testosterone, LH, and FSH serum levels might be ascribed to 1) hypothalamus and pituitary tissue damage and 2) decreased Leydig cells activity and Sertoli cells function. Also, our findings revealed an enhancement in serum concentrations of GnRH, FSH LH, and testosterone in SE and EM/SE groups. In agreement with our results, Lukaso (2017) claimed that orally-administered selenium increased LH and testosterone hormones (38). These hormonal changes may be due to a reduction in oxidative stress (12). On the other hand, selenium may activate the GnRH receptor (in the anterior pituitary), thereby, increasing the production of LH and FSH. These hormonal changes might be due to reduced oxidative stress (12) and the effect of selenium on the activation gland, resulting in increased LH and FSH production in Se and EM/SE groups (38). Leptin, an adipocyte-derived hormone, has a positive effect on normal male reproductive system activity, but when present in excess, it can have detrimental effects influence on the male reproductive system (39) and inhibit steroidogenesis in the testis (40). Our findings showed that EMF increased the serum leptin levels. Although the precise mechanism by which leptin activates the GnRH neurons to act on the reproductive system remain unknown, in 2012 SY Ahn showed that extra leptin in serum levels could cause a reduction in GnRH levels which subsequently lead to the decrease of GnRH, LH, and FSH hormones. It seems that excess leptin might be inhibited the KiSS-1 expression in anteroventral periventricular nucleus (AVPV) of the hypothalamus and their G-protein-coupled receptor (KiSS-1R) that has been recognized in the hypothalamus and pituitary gland (41).

Moreover, previous studies have paid little attention to the direct effect of electromagnetic field on leptin; however, some research results have revealed that electromagnetic radiation leads to more ACTH secretion, and cortisol level is increased by the adrenal gland, which is closely associated with leptin secretion (42, 43). 

The present study results showed that the serum leptin level was reduced following selenium administration in the EM/SE group. According to some scientific reports, there is an inverse relationship between the level of blood leptin and selenium. In this line, Kim CY (2019) showed that selenite prevented adipogenesis. As previously stated, adipose tissue is the main source of leptin secretion. Therefore, by preventing adipogenesis, selenium indirectly reduces the amount of leptin in serum. On the other hand, testosterone is also capable of preventing leptin secretion by controlling the white adipose tissue, resulting in inverse correlation between leptin and testosterone levels. The present results also showed that the electromagnetic field increased the leptin receptor while prescription selenium reduced leptin receptor in testicular tissues. Leptin receptor, as an adipocyte receptor specific for the leptin hormone, is located on the cell membrane, and it has an extracellular, transmembrane, and intracellular domain. Increased leptin receptor ensues** sperm dysfunctions (44).

Aromatase is an enzyme responsible for a key step of estrogen biosynthesis and plays a pivotal role in sexual maturation. The imbalance of aromatase in the serum can cause problems in the male sexual cycle. As mentioned, aromatase can convert testosterone to estradiol in the final stage, and estrogen enhancement leads to spermatogenesis defect. Furthermore, this hormone controls the luminal fluid reabsorption in the epididymis (45, 46). In the current research, electromagnetic radiation raised serum aromatase level. It seems that electromagnetic field increases oxidative stress, which decreases the amount of testosterone and increases estrogen levels by increasing aromatase (47). In the present study, administration of selenium reduced the serum aromatase level. Similarly, it has been reported that selenium, as an antioxidant, reduces the aromatase level and increases testosterone (48).

Our findings indicated that electromagnetic radiation reduced the germinal epithelium thickness and the seminiferous tubule diameter. According to previous studies, electromagnetic fields damage seminiferous epithelia as well as Leydig cells (49). However, Hunci claimed that smartphone radiation was not able to change the diameter of the seminiferous tubule (50). According to the study of Wang, electromagnetic radiation might increase the permeability of the blood-testis-barrier and bloodstream-containing free radicals, leading to increased germ cell death and apoptosis (51). The previous studies confirm our results in which selenium consumption increased the diameter of the seminiferous tubules (52).

Additionally, the electromagnetic fields induced apoptosis in spermatogenic cells (spermatogonia, primary spermatocytes, and spermatid). In some studies, such as Even’s, all frequencies generated by electromagnetic fields were able to impact the process of apoptosis. It has been proposed that electromagnetic field may cause DNA fragmentation or mitochondria damage in spermatogenic cells, ensuing apoptosis induction. The present study revealed that selenium significantly reduced apoptosis. It seems that selenium can improve the antioxidant capacity of cells and protect against the adverse effects of EMF (53-55).

 The number of sperm was further observed to be reduced and abnormal sperm were increased in electromagnetic field-exposed animals. Reduced sperm might be ascribed to the localized heat generated by electromagnetic radiation or damaged Sertoli cells as a result of changes in the sperm cell cycle (56, 57). The results of this research also showed that selenium prescription was able to significantly increase the number of sperm and cause a significant reduction in the percentage of sperm abnormality. Certain researches have adduced to the finding of the present study (58). Other research results showed that selenium as a supplement increased the quality and quantity of sperm (59).

## Conclusion

Exposure to EMF 2 hr/day within 16 days reduced serum LH, FSH, testosterone, GnRH, and leptin levels. Moreover, EMF was able to reduce the diameter of seminiferous tubules and germinal epithelium thickness and increase spermatogenic cell apoptosis and leptin receptor’s overexpression. In addition, EMF was able to reduce the sperm count and increase the unusual sperm in the epididymis of rats. Finally, the results of the present research indicate serum selenium level may protect against the side effects of electromagnetic fields on testicular tissue, sex hormones, leptin receptor expression, and sperm parameters in rats.
